# Dielectric barrier discharge plasma microbubble reactor for pretreatment of lignocellulosic biomass

**DOI:** 10.1002/aic.16212

**Published:** 2018-09-07

**Authors:** Alexander Wright, Hemaka Bandulasena, Christopher Ibenegbu, David Leak, Thomas Holmes, William Zimmerman, Alex Shaw, Felipe Iza

**Affiliations:** ^1^ Dept. of Chemical Engineering Loughborough University Loughborough Leicestershire, LE11 3TU U.K; ^2^ Dept. of Biology and Biochemistry University of Bath Bath, BA2 7AY U.K; ^3^ Dept. of Chemical and Biological Engineering University of Sheffield Sheffield, S10 2TN U.K; ^4^ Wolfson School of Mechanical, Electrical and Manufacturing Engineering Loughborough University Loughborough Leicestershire, LE11 3TU U.K.

**Keywords:** microbubbles, DBD plasma, ozone, OH radicals, miscanthus, biomass

## Abstract

A novel lignocellulosic biomass pretreatment reactor has been designed and tested to investigate pretreatment efficacy of miscanthus grass. The reactor was designed to optimize the transfer of highly oxidative species produced by dielectric barrier discharge plasma to the liquid phase immediately after generation, by arranging close proximity of the plasma to the gas‐liquid interface of microbubbles. The reactor produced a range of reactive oxygen species and reactive nitrogen species, and the rate of production depended on the power source duty cycle and the temperature of the plasma. Ozone and other oxidative species were dispersed efficiently using energy efficient microbubbles produced by fluidic oscillations. A 5% (w/w) miscanthus suspension pretreated for 3 h at 10% duty cycle yielded 0.5% acid soluble lignin release and 26% sugar release post hydrolysis with accelerated pretreatment toward the latter stages of the treatment demonstrating the potential of this approach as an alternative pretreatment method. © 2018 The Authors. AIChE Journal published by Wiley Periodicals, Inc. on behalf of American Institute of Chemical Engineers. © 2018 The Authors. AIChE Journal published by Wiley Periodicals, Inc. on behalf of American Institute of Chemical Engineers. *AIChE J*, 64: 3803–3816, 2018

## Introduction

Rising concern over depletion of fossil fuel reserves and greenhouse gas emissions from fossil fuel combustion has led to a high level of interest in biofuel and commodity chemical production from renewable resources. Currently, the top producers of bioethanol (US and Brazil) use food crops such as corn and sugarcane as the main raw material.^1^ However, use of food sources for biofuel production will not be sustainable in the current climate as most countries are struggling with feeding their population.^2^ Additionally, current bioethanol production is only a small fraction of the total worldwide fossil fuel consumption. As a result, research efforts are now shifting to lignocellulosic biomass as a renewable source that does not compete with food production.

Lignocellulosic biomass is the most abundant form of renewable energy and can be used for liquid biofuel production. Ninety percent of the dry weight of lignocellulosic biomass is made up of cellulose, hemicellulose, and lignin where cellulose and hemicellulose can be fermented to form bioethanol.^3^ *Miscanthus* x *giganteus* (miscanthus), a temperate perennial grass, has a high carbohydrate content of 40.6‐46.4% cellulose and 28.8–29.2% hemicellulose,^4^ but also contains 19.3–31% lignin.^5^ These biomass components are strong and flexible composite polymers, which serve to maintain the structural integrity of the plant.^6^ Among perennial grass plants, miscanthus is known to have the highest biomass yield,^7^, ^8^, ^9^, ^10^, ^11^ and is well known to adapt to various agronomic conditions making it a suitable candidate for this study. The chemical composition and physical structure of lignocellulose is constructed to protect cellulose from degradation, leading to biomass recalcitrance.^12^ Ethanol production from lignocellulosic biomass through biological conversion is a process that typically includes biomass pretreatment, production of monomeric sugars from the pretreated biomass by hydrolysis of the hemicellulose and cellulose polysaccharides, and ethanol production by fermentation of these sugar monomers.^13^ The greatest barrier that limits the use of lignocellulose as an industrial feedstock lies in its indigestibility; hence physical/chemical pretreatment is needed to make the cellulose more accessible for enzymatic hydrolysis.^14^ The pretreatment methods can be broadly classified as physical,^15^ chemical,^16^ and biological.^17^ Pretreatment provides access to hydrolytic enzymes by increasing accessible surface area, decrystallizing cellulose, removing hemicellulose, and altering the structure of lignin.^18^

One of the most effective methods of pretreatment is steam explosion,^19^ where biomass is heated to between 160 and 220°C with steam at pressures of 0.52–2.25 MPa. The steam impregnates the biomass and is rapidly (explosively) released by decompression breaking down the biomass. This process works by physically and chemically modifying the biomass so that lignin, hemicellulose and cellulose are separated and the latter can be easily hydrolyzed by enzymes and fermented by microorganisms.^20^ Although steam explosion is effective, it is highly energy intensive. Therefore, it would be useful to develop alternative pretreatment methods that are effective as well as efficient to make biofuel production economically feasible. In this study, we report a novel lignocellulosic pretreatment reactor that separates lignin using reactive species produced by dielectric barrier discharge (DBD) plasma and compare the efficiency of treatment with steam explosion.

Besides steam explosion, ozonolysis has been used effectively to reduce lignin and hemicellulose in a wide range of lignocellulosic materials such as wheat, rye, and grass.^21^ Ozone (O_3_) is a powerful oxidant with an oxidation potential of 2.07 V that can cleave carbon‐carbon double bonds that are abundant in lignin to form aldehydes via the well‐known process of ozonolysis, as seen in reaction Scheme 1.^22^, ^23^ Although this has been identified as a key reaction, lignin is a highly complex molecule with multiple functional groups including ester and ether linkages which can also be attacked by other reactive species.

As highlighted by Rosenfeldt et al.,^24^ an alternative to O_3_ pretreatment is to use a combination of oxidative species. The combination of O_3_, UV and hydroxyl radicals (^•^OH) has been widely reported as an advanced oxidation processes (AOPs) for applications such as water and wastewater treatment,^24^ but their use in lignocellulosic biomass pretreatment is limited. New technologies for advanced oxidation processes are emerging and the question remains whether this could become a feasible alternative solution for pretreatment of lignocellulosic biomass.

In this study, we hypothesize that biomass pretreatment can be effectively performed using the combined effect of O_3_, UV, ^•^OH, and other reactive oxygen and nitrogen species produced by an atmospheric pressure air plasma. A DBD plasma was chosen as the plasma source for their relative simplicity, robustness, and proven scalability.^25^ The cocktail of reactive species generated in the plasma will mainly depend on the electrical parameters used to generate the plasma, but also on the background gas used. For plasmas generated in humid air, reactive oxygen species (ROS) such as O_3_, ^•^OH, and hydrogen peroxide (H_2_O_2_) are produced in addition to reactive nitrogen species (RNS) such as NO_2_, NO_3_, HNO_3_, and N_2_O_5_.^26^, ^27^ In addition to these chemical species, plasma also emits UV light that could take part in photochemical reactions at the gas‐liquid interfaces. Most notably, UVC which breaks down hydrogen peroxide and O_3_ to form ^•^OH.^28^, ^29^, ^30^

In summary, pretreatment is one of the most expensive processes of converting lignocellulosic biomass to biofuel as highlighted by Mosier et al.,^18^ an area where significant improvements can be made. The main purpose of this study was to investigate the effectiveness and efficiency of a novel lignocellulosic pretreatment reactor that is designed to produce and transfer reactive species in situ using DBD plasma and energy efficient microbubbles. This article is organized as follows. In design features section, design features of the plasma microbubble reactor are described and how various technologies applied in this approach aid the pretreatment process is presented. In materials and methods section, reactor characterization experiments and biomass pretreatment tests are described. In results and discussion section, results on the reactor performance and effectiveness of pretreatment is discussed. In conclusion section, conclusions are drawn.

## Design Features

The following key features were incorporated into the reactor design to intensify biomass pretreatment and to make the process efficient.

### *Electrodes and dielectrics*

**Figure 1:**
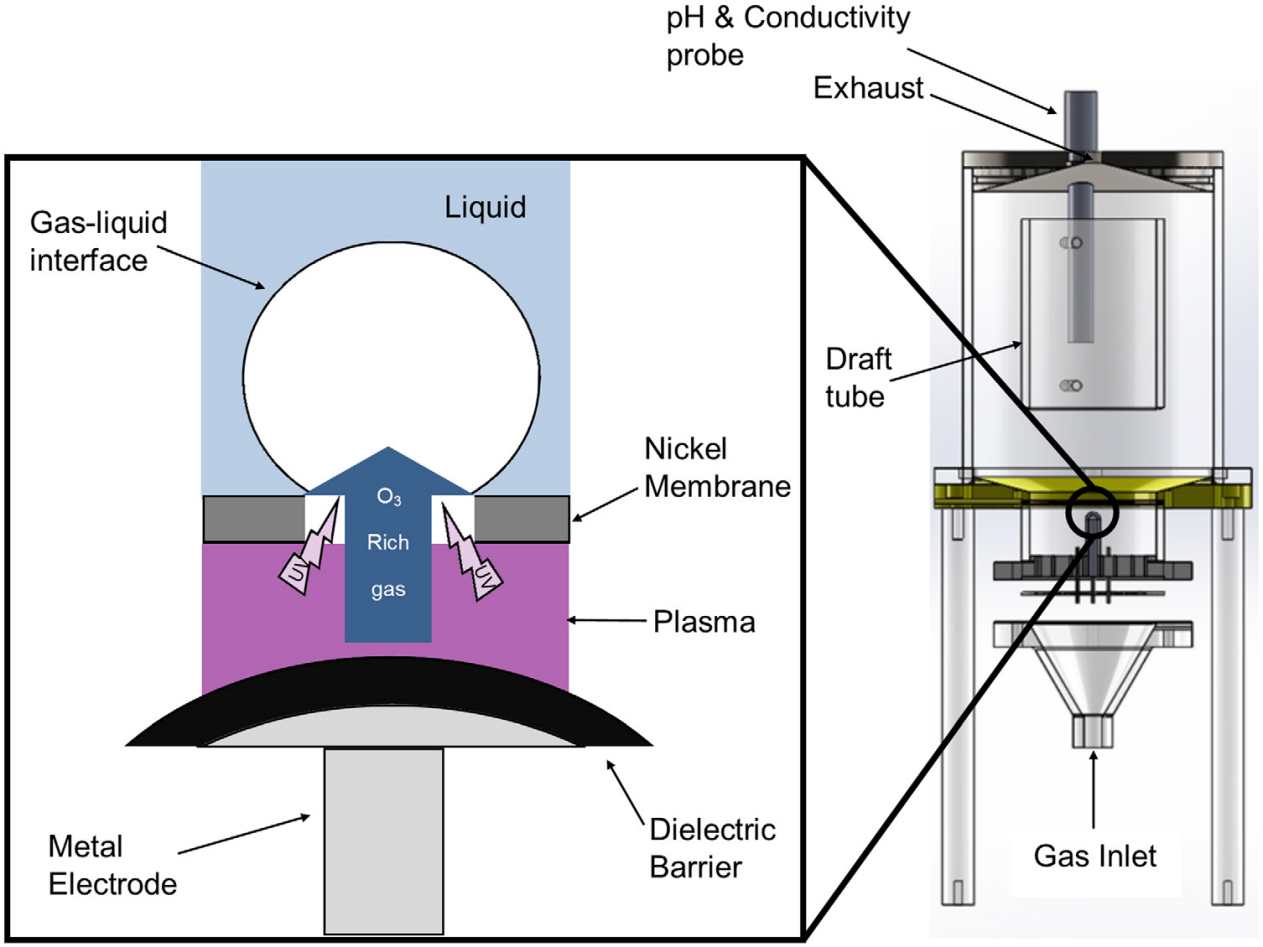
A schematic diagram of the DBD plasma at the gas‐liquid interface. ROS, RNS, and UV light produced *in situ* at the microbubble interface making the transfer of short‐lived species to the liquid possible. [Color figure can be viewed at http://wileyonlinelibrary.com]

The reactor incorporates a DBD plasma module which consists of two electrodes (see Figure 1). The microporous nickel membrane that is used to produces microbubbles serves as the ground electrode. The second electrode consists of 37 identical stainless‐steel rods of 5 mm in diameter encased in hollow alumina tubes (Almath Ltd, UK) of 10 mm in diameter with a wall thickness of 1.25 mm. These tubes were positioned below the nickel membrane to form a gap of approximately 1 mm between the electrodes. The spacing between the alumina tubes was determined to allow sufficient gas flow to the plasma chamber and to provide a cooling effect. The vertical position of each metal rod encased in alumina tubes can be adjusted individually to achieve a uniform plasma discharge. This is found to be an essential adjustment to compensate slight variations of the dielectric thickness and to account for the complex shape of the nickel membrane under liquid loading. Alumina was chosen as the dielectric material as it possesses a high dielectric constant, high thermal conductivity, and can withstand high temperatures without cracking.

### *Proximity of plasma to the gas‐liquid interface*

Figure 1 shows a schematic diagram of the reactor configuration highlighting how the plasma module fits at the bottom of the reaction tank. This configuration allows close proximity of the plasma to the gas‐liquid interface facilitating immediate species transport at production and allowing UV emission to reach a thin liquid shell around the microbubbles.

As most chemical species produced by DBD plasmas are highly reactive and short lived, it is important to locate the plasma close to the gas‐liquid interface to facilitate efficient transfer of those species produced to the liquid phase. For instance, ^•^OH have a half‐life of approximately 10^−9^ s 31 and should be produced in the liquid phase or at gas‐liquid interfaces for the maximum effect. It has been shown that radicals such as ^•^OH and O_2_^−^ can be delivered to a liquid from a plasma.^32^, ^33^, ^34^ It is also possible to form ^•^OH from dissolved O_3_ by irradiating with UV.^24^ Bruggeman and Schram provided a reaction scheme for ^•^OH radical formation for this case.^35^ This reactor combines and intensifies the above mentioned techniques by producing the plasma at the gas‐liquid interphase. Table 1 shows several species produced by the plasma in comparison with other species with a high oxidation potential.

**Table 1:** A Comparison of the Oxidation Potential of Species Produced Within This Study^32^

Species	Oxidation Potential (*V*)	Produced in this Study
F_2_	3.03	N
^.^OH	2.80	Y
O	2.42	Y
O_3_	2.07	Y
H_2_O_2_	1.78	Y
HO_2_	1.70	Y
Cl_2_	1.36	N

### *Microbubbles for enhanced mass transfer and mixing*

The multiphase mass transfer from gases to liquids significantly improves when microbubbles are used as they provide a high surface to volume ratio.^36^ There are a number of ways to produce microbubbles including supersaturated liquid release through a specially designed nozzle^37^ and ultrasound.^38^ However, these methods are highly energy intensive and would increase the cost of biofuel production. Zimmerman et al.,^39^ reported an energy efficient method for production of microbubbles using fluidic oscillation and demonstrated significant improvements in mass‐transfer rates.^39^, ^40^, ^41^ This method incorporates a fluidic oscillator in conjunction with a microporous membrane sparger that breaks off the bubbles when they are in hemispherical cap, that is, when they are at their smallest size during growth. A detailed review of this microbubble generation method can be found in Refs. 39, 40, 42. This method has been employed in this study to improve the mass‐transfer rates from the plasma to the pretreatment liquid and thereby the performance of the reactor.

The fluidic oscillator used in this study was a pilot‐scale unit that require a threshold air flow rate of approximately 80 slpm to operate consistently. However, this flow rate exceeds the gas input required for an 80 mm diameter membrane sparger. Therefore, only a fraction of the outlet air stream from the oscillator (∼2 slpm) was used for microbubble production. A nickel membrane (Micropore Ltd, UK) with a mean pore size of 20 μm with a pitch of 180 μm was selected as the sparger membrane. These membranes are naturally hydrophobic and, if untreated, large bubbles will be produced.^43^ Therefore, membranes were treated chemically prior to their use to achieve a hydrophilic membrane surface. The optimal conditions for microbubble production was found to be 5 m feedback loop length and an inlet air flow rate of 160 slpm to the fluidic oscillator, which corresponds to an oscillation frequency of 40 Hz. These conditions were predetermined from a preliminary study using a high‐speed camera and ImageJ analysis. The average bubble diameter achieved was 385 μm with a coefficient of variation of 20%.

### *Airlift loop for biomass suspension and improved mixing*

In addition to improving the mass‐transfer rate, it is vital to keep the biomass well suspended and well mixed to treat the batch homogeneously. This mixing process can be promoted by introducing a draft tube to the core of the reactor with a swarm of microbubbles. As highly dense cloud of microbubbles rise through the inner draft tube, convection currents are generated throughout the reactor promoting mixing.^44^ Airlift reactors have been proven to improve mass‐transfer rates and to allow low gas flow rates to be used while allowing high gas retention.^36^ The use of an airlift bioreactor also eliminates the need for mechanical mixing, decreasing the cost of operation.^45^ The maximum volume of the reactor was designed to be 3 L to test a sufficient sample, but not too large to waste limited miscanthus samples available. The height (*H*) and the diameter (*D*) of the reactor was chosen to be 200 mm and 152 mm, respectively, to achieve a D/H ratio of ¾ suitable for airlift loop convection flow. The draft tube height and the diameter were chosen to be 120 mm and 80 mm, respectively. All dimensions were calculated according to,^36^ and the mixing induced by a swarm of bubbles rising within the draft tube led to high mass transfer rates of plasma products as demonstrated by Rehman et al.^41^

### *Design of the power supply*

The electrical discharge was driven by a custom‐built full‐bridge resonant power supply that delivered a sinusoidal voltage of 16.4 kV_RMS_ at 21.2 kHz. To control the gas temperature of the plasma, the sinusoidal signal was modulated on and off at regular intervals. In this study, the duty cycle (Eq. (2)) was varied between 5% and 45% by adjusting the “off” time between 122 ms and 1900 ms while keeping the “on” time constant at 100 ms.

## Materials and Methods

### *Experimental setup*

**Figure 2:**
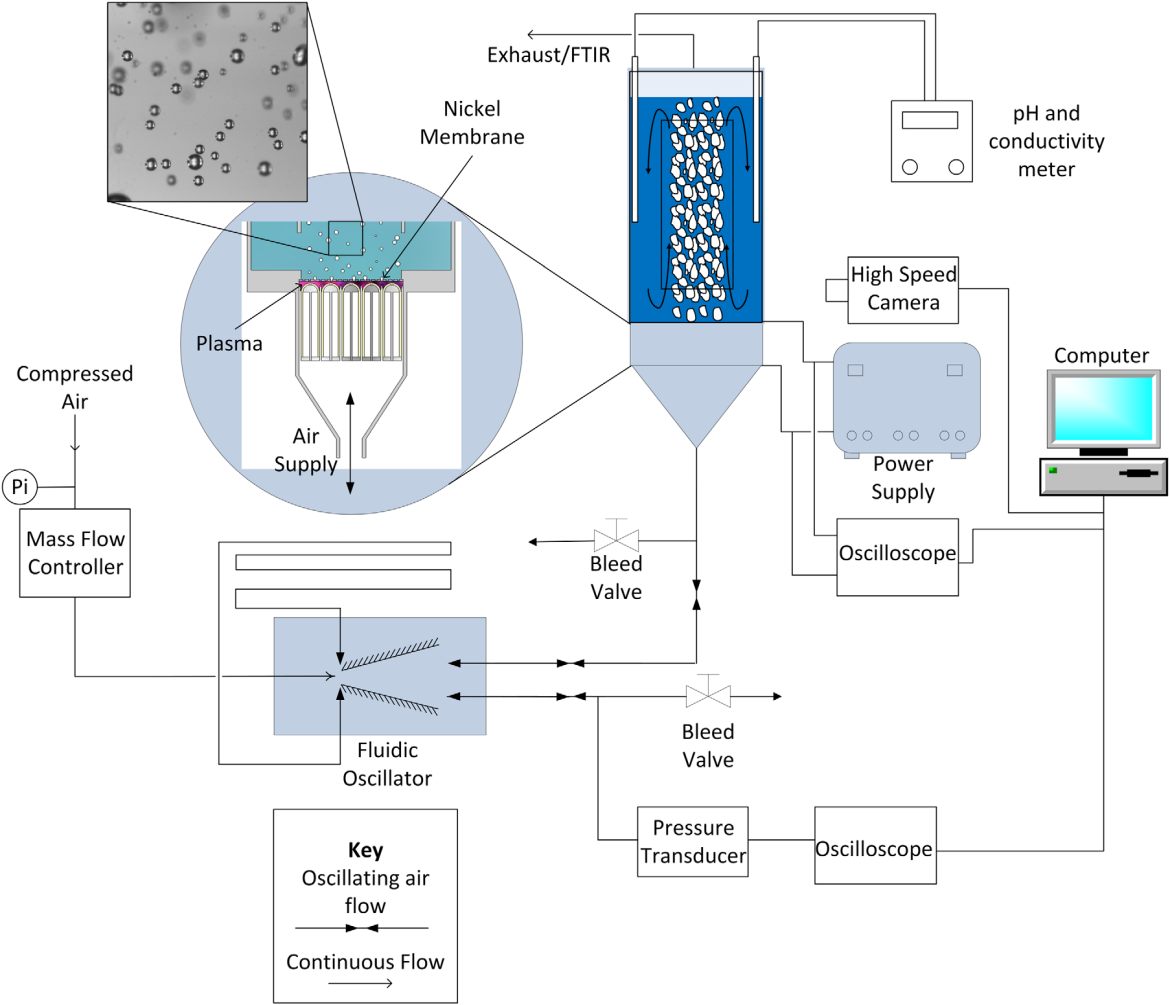
Experimental setup for the pretreatment reactor with microbubble generation. [Color figure can be viewed at http://wileyonlinelibrary.com]

The experimental setup is shown in Figure 2. The setup consists of a mass flow controller, a fluidic oscillator and the plasma microbubble reactor as described in materials and methods section. Filtered compressed air was supplied to the fluidic oscillator at 160 slpm via a mass flow controller (Alicat, MCR 500), and the fluidic resistances on the two oscillator outlets were balanced using pinch valves for symmetric operation. A small proportion of the oscillating gas flow, ∼2 slpm, from an outlet from the fluidic oscillator was connected to the bottom of the reactor while releasing the excess gas to the atmosphere. Such a large proportion of gas was released to the atmosphere in this case as a pilot‐scale fluidic oscillator was used in this study. The gas that enters the reactor then flows in between alumina tubes and reaches the plasma generation zone. The plasma was ignited between the top of the alumina tubes and the membrane by a custom‐built external power supply. O_3_ and other reactive species generated by the plasma were then dispersed in the liquid by microbubbles produced on the membrane surface.

The setup was instrumented for monitoring and measurement of several important quantities. A high‐speed camera (Photon Fastcam, M2.1) coupled with a long‐distance lens (Infinity, KC) were placed level with the nickel membrane to measure the microbubbles produced. The bubble size distribution was determined by analyzing acquired images with ImageJ software. pH, conductivity, and liquid phase temperature were measured using a combined meter (Thermo Fisher Scientific Inc, Orion star A215) positioned in the mid down‐comer region of the reactor. An oscilloscope (Tektronix, TDS 2012 B) and a high voltage probe (Tektronix P6015) were connected to the power supply to measure the amplitude, frequency, and duty cycle of the applied voltage. A separate oscilloscope (Pico technology Ltd, PicoScope 6402C) was connected to a pressure transducer (Hydrotechnik UK Ltd, PR140 HT‐PD) in‐line with the output feed of the fluidic oscillator to measure frequency of the oscillating gas supply. The exhaust gas from the reactor was fed to a gas cell (1–16 M long pass, Pike Technologies) that was connected to a Fourier transform infrared spectrometer (Jasco FTIR‐4700LE) to measure the outlet gas composition. To monitor the gas phase temperature in the plasma module, a k‐type thermocouple (TFC, 307P) was used. Thermocouple measurements are only possible when the plasma is turned off due to electrical interference caused by the plasma, and therefore the readings could have been slightly lower than the maximum value reached during operation. A spectrometer (Andor, SR‐303i‐A) was used to characterize the emission spectrum from the plasma. An optical fiber (Ocean optics Inc, QP600‐1‐UV‐VIS) was inserted through a port in the plasma module wall and directed at the plasma to collect the emission spectra.

The frequency of the AC power supply was tuned by visually observing the plasma for a uniform distribution over the entire membrane. The optimum frequency was determined to be 21.2 kHz, and the frequency of operation was kept constant throughout the experiments. The power supply was operated at duty cycles of 5%, 10%, 15%, and 45%, and the time averaged power delivered to the plasma was determined by analyzing charge vs. voltage (*Q*‐*V*) Lissajous diagrams.^46^

Prior to each pretreatment experiment, the nickel membrane was first sonicated in 4 M sodium hydroxide (Fisher Scientific UK Ltd.) for 10 min. The membrane was then rinsed with DI water followed by a further 10 min sonication in 2 M citric acid (Sigma Aldrich Co. LLC) to reduce the contact angle from 103.5° ± 3.7° to 26.7° ±3.9°. The reactor was then assembled, and the electrodes encased in alumina tubes were adjusted to give uniform plasma coverage under the membrane surface.

### *Measurement of ROS and RNS*

First, the reactor was run without any liquids to characterize the plasma species generated, using Fourier transform infrared spectroscopy (FTIR). O_3_ concentration at the exhaust was determined by IR absorbance at 2122 cm^−1^, and a range of RNS species with the following absorbing bands were also detected: N_2_O at 2240 cm^−1^, N_2_O_5_ at 750 cm^−1^, and HNO_3_ at 890 cm^−1^.^47^

The transfer of oxidative species from gas to liquid was measured using specific chemical probes. To measure the combined effects of the oxidative species including O_3_, ^•^OH, and H_2_O_2_ in the liquid phase, the chemical probe Potassium indigo carmine (indigo) was used (see reaction (3)).

The procedure described in Ref. 48 was performed by creating 2.8 L of known concentration of indigo along with a phosphoric acid and sodium dihydrogen phosphate as the buffer (Sigma Aldrich Co. LLC). Measurements were taken in situ with a halogen light source (HL‐2000, Ocean Optics) directed through a port on the reactor with an optical fiber (QP600‐1‐XSR, Ocean Optics) coupled with a collimating lens and transmitted light collected at 180° to the first with a spectrometer (HR2000+, Ocean Optics). The concentration of indigo could then be calculated using the Beer‐Lambert law.^48^

To measure the occurrence of ^•^OH specifically, terephthalic acid was used as described by Mason et al.,^49^ When terephthalic acid reacts with ^•^OH, a fluorescent product, 2‐hydroxyterephthalic acid (HTA) is produced as shown in reaction (4). The fluorescence emitted by HTA at 425 nm due to excitation at 315 nm (light source: LLS‐315, Ocean Optics) was measured using spectrometer (HR2000+, Ocean Optics).

### *Miscanthus pretreatment*

*Miscanthus* x *giganteus*, sourced from Wiltshire, UK, was air‐dried to 11.3% moisture content. First, miscanthus samples were milled in a cutting mill (SM2000, Germany) for 5 min followed by sieving (20–80 mesh) to reduce the stem size from 3–5 cm to 180–850 μm range. The prepared miscanthus samples were then treated in the plasma microbubble reactor as dilute solutions or as a fluidized bed in dry form. Even though dry treatment of lignocellulosic biomass using ozonolysis has been reported earlier,^21^ pretreatment in the vicinity of a DBD plasma with a range of reactive products has not been reported elsewhere. The current tests, therefore allow comparison of wet vs. dry treatment to assess the virtues and limitations of conditions used.

For wet pretreatment, dilute miscanthus samples of 0.4%, 5%, 8%, 10%, 15%, and 50% (wt/wt) were prepared by mixing the required amount of milled miscanthus in deionized water for 1 h. This ensured homogenous solutions and provided sufficient time for biomass to swell and open up the structure for reactive species to penetrate. The duty cycle of the plasma was kept constant at either 10% or 45%, and the treated samples were taken at 1 h intervals for up to 4 h. For dry pretreatment, 60 g of milled miscanthus was suspended above the membrane using oscillatory flow. During preliminary experiments, it was observed that constant air flow causes channeling in dry miscanthus beds due to fibrous nature of the grass causing uneven distribution of gaseous products from the plasma. However, the oscillatory flow provided by the fluidic oscillator avoided flow channeling by constantly vibrating the biomass and rearranging the packing orientation. To compare this novel plasma microbubble approach with steam explosion, 70 g of miscanthus was also treated in a 1 L high pressure reactor (Parr instruments) connected to a steam generator (Prioclave, UK) for a period of 10 min at 185°C and 0.85 MPa followed by ice cooling.

### *Composition analysis*

Wet ozone pretreated samples were initially freeze dried prior to compositional analysis. The moisture content of raw and pretreated miscanthus samples was determined by drying overnight at 105°C in a hot air oven. Carbohydrate composition of miscanthus was analyzed following acid hydrolysis.^50^, ^51^ First, the samples were hydrolyzed with 3 mL of 72% w/w H_2_SO_4_ and incubated at 30°C for 1 h with shaking at 100 rpm. Then the samples were diluted with 84 mL of Milli‐Q water, autoclaved at 121°C for 60 min, cooled to room temperature and neutralized with calcium carbonate (CaCO_3_) to between pH 6.0 and 7.0. The samples were clarified by filtration through a 0.22 mm nylon membrane filter and kept at −20°C prior to analysis. A sugar recovery test was applied to the samples to compensate for sugar loss/decomposition due to acid hydrolysis.^52^, ^53^ The carbohydrate composition in each sample was quantified by ion chromatography taking into consideration the moisture content.

### *Enzymatic hydrolysis*

Enzymatic hydrolysis solution (20 mL total volume) contained 2.44% (w/w) miscanthus adjusted to pH 5.0 with 5M solution of H_3_PO_4_ or KOH, an enzyme mixture containing 7.5–30 filter paper units (FPU)/g glucan of Cellic CTec2 cellulase (Novozymes) and 100 U/g of SEB xylanase (Advanced Enzymes technology LTD). The reaction was incubated at 50°C for 72 h with shaking at 250 rpm before terminating by boiling at 100°C for 5 min.^53^ All chemicals, reagents and equipment in this step were sterilized before use. The miscanthus hydrolysate was centrifuged, filtered and analyzed for total sugars (monomeric and oligomeric sugars) using the NREL methods.^50^, ^51^

### *Analysis of sugars by high performance liquid chromatography*

The amount of sugars in all samples were analyzed in an Agilent HPLC system (Agilent Technologies, Santa Clara, CA) equipped with a refractive index and UV detector. Separation was performed on a Phenomenex Rezex RHM Monosaccharide H column (300 m × 7.8 mm, Phenomenex Inc, Torrance, CA) at 65°C for 30 min with a flow rate of 0.6 mL/min and 5 mM H_2_SO_4_ as the mobile phase.^53^ To determine the effect of washing prior to enzymatic hydrolysis, wash water was also hydrolyzed to determine the sugar content.

### *Acid soluble lignin and total lignin*

Total lignin content was determined by a two‐step acid hydrolysis method according to a laboratory analytical procedure of the National Renewable Energy Laboratory (NREL).^50^, ^51^ The acid soluble lignin was analyzed by initially freeze drying the samples to ∼95% dry matter followed by milling and oven drying overnight at 105°C. Then acid hydrolysis was performed as described earlier. Following acid hydrolysis, biomass was filtered and analyzed using a UV spectrophotometer at 205 nm with an absorptivity constant of 110 L/g/cm for ASL from miscanthus.^54^ A sample of 3% sulphuric acid in MilliQ water was treated the same way as the miscanthus samples and used as blank and diluent. Moisture content was determined by oven‐drying overnight at 105°C. The ASL was calculated using Eq. (5), where *A* is the absorption value at 205 nm, *D* is the dilution ratio of the sample, *V* is the sample volume, *K* is the absorptivity constant for miscanthus lignin, and *W* is the weight of biomass at the start of ASL analysis.

The acid‐insoluble lignin was calculated gravimetrically after correction for ash according to the NREL standard laboratory analytical procedure (LAP).^50^, ^51^ For this, the solids remaining on the filter crucible were washed three times with hot distilled water, oven dried at 105°C overnight and weighed together with the crucible. The weight of the crucible and dry residue was recorded (WB). Then, the residue was ashed in the muffle furnace at 200°C for 30 min and 575°C for 4 h. The crucibles and ash were weighed as (WC). Acid‐insoluble lignin (AIL) in the original sample was then calculated as: AIL (%) = (WB‐WC) × 100/*W*%. Total lignin was calculated as ASL + AIL.

### *Scanning electron microscopy*

Scanning electron microscopy (SEM) images of miscanthus samples before and after pretreatment were taken to provide qualitative morphological analysis in addition to the chemical analysis of acid soluble lignin and sugars released. These images should provide visual confirmation of any structural changes to lignocellulosic biomass following pretreatment. The samples were dried and coated with gold palladium using a sputter coater (SC7640, Quorum Technologies Ltd) for 180 s at 25 mA for imaging by scanning electron microscopy (LEO 1530VP, Carl‐Zeiss).

## Results and Discussion

### *Plasma gas analysis*

**Figure 3:**
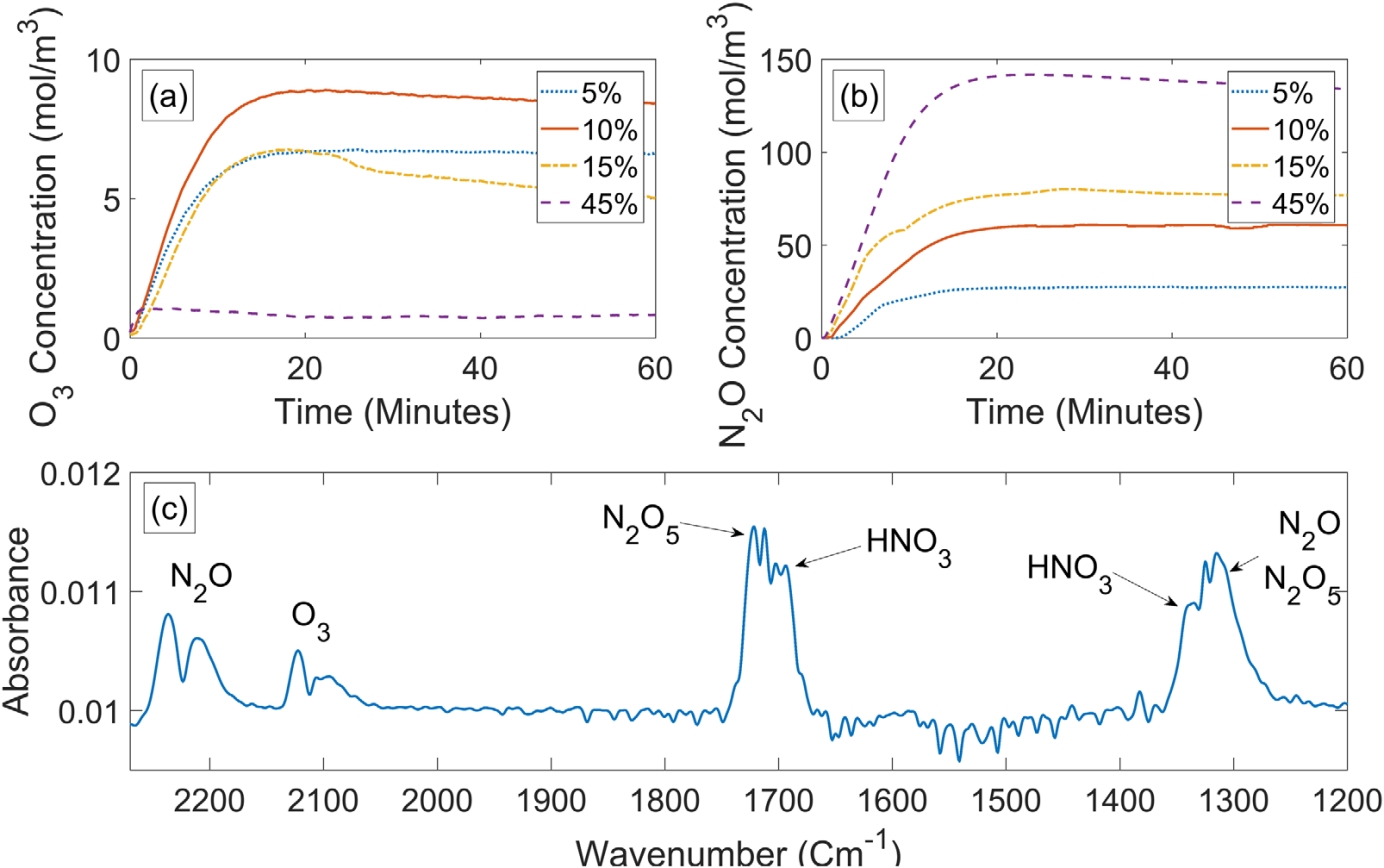
Long lived ROS and RNS species measured by FTIR for the empty reactor (a) O_3_ (b) N_2_O (c) RNS. [Color figure can be viewed at http://wileyonlinelibrary.com]

The concentration of various gaseous products of the DBD plasma from the empty reactor for duty cycles varying from 5% to 45% is shown in Figure 3. For lower duty cycles of 5%, 10%, and 15%, the O_3_ concentration increases for ∼15 min and reaches its maximum value followed by a gradual drop in concentration Figure 3a. In contrast, the higher duty cycle tested (45%) produced a low level of O_3_ during the 60 min run. This is attributed to the increase in gas temperature as the input power increases with the duty cycle. Ozone production is favored at low temperatures,^25^ hence the drop in O_3_ concentration is more pronounced at 45% duty cycle as the plasma module reached ∼195°C after 60 min of operation compared to ∼46°C observed for the 10% duty cycle. The time to reach the maximum O_3_ concentration depends on the O_3_ generation rate, the thermal evolution of the reactor and the time required to fill the FTIR gas cell. Similar maximum concentrations of ozone were recorded for 5% and 15% duty cycles as the potentially higher production rate expected at the larger duty cycle were suppressed by the higher temperatures reached at 15% duty cycle. The steeper drop in O_3_ concentration observed for 15% duty cycle after ∼20 min confirms this conjecture. However, it must be noted that high duty cycles can produce O_3_ at a higher rate, if sufficient cooling can be provided to the plasma module externally. O_3_ concentration up to 8.9 × 10^−3^ mol/L (426 ppm) observed for 10% duty cycle greatly surpasses the value of 8.33 × 10^−4^ – 1.21 × 10^−3^ mol/L (40–58 ppm) reported by Panneerselvam et al., that successfully achieved 51% delignification of miscanthus.^55^ It is anticipated that when liquid is introduced to the reactor, the ground electrode will be cooled by convection currents above the membrane and the O_3_ generation can be maintained for longer periods at higher duty cycles. As ozone is one of the main reactive species capable of delignification of miscanthus, 10% duty cycle was selected as one of the test conditions for the miscanthus pretreatment experiments.

The concentration of RNS produced by the reactor during a 60‐min run is shown in Figures 3b, c. The concentration of N_2_O increases initially and reaches a plateau after ∼20 min for all the duty cycles tested. The maximum concentration of N_2_O produced for each case depended on the duty cycle used, that is, high duty cycles produced a higher level of N_2_O compared to the lower duty cycles tested as part of the O_3_ produced by the plasma is converted to N_2_O by a series of pathways, many of which depends on the thermal evolution of the reactor.^56^ Figure 3c shows various RNS species produced by the plasma such as HNO_3_, N_2_O_5_, and N_2_O for a 10% duty cycle experiment, which could also contribute to the pretreatment process. Other RNS species such as NO and NO_2_ will also be generated by the plasma under these conditions, but the concentrations were too low be to detected downstream by FTIR measurements.^27^, ^56^ Further analysis of Figure 3 suggests that there are two operating regimes. First, low duty cycles that favored O_3_ production with optimum conditions at approximately 10% duty cycle. The second regime showed higher concentrations of RNS and low O_3_ concentrations at higher duty cycles of ∼45%. The species concentrations shown in Figure 3 are characteristics of the empty reactor under convective cooling provided by the incoming air flow used for microbubble production. These operating conditions and the product concentrations can be shifted, if the plasma operates under different cooling conditions.

### *Spectral analysis*

**Figure 4:**
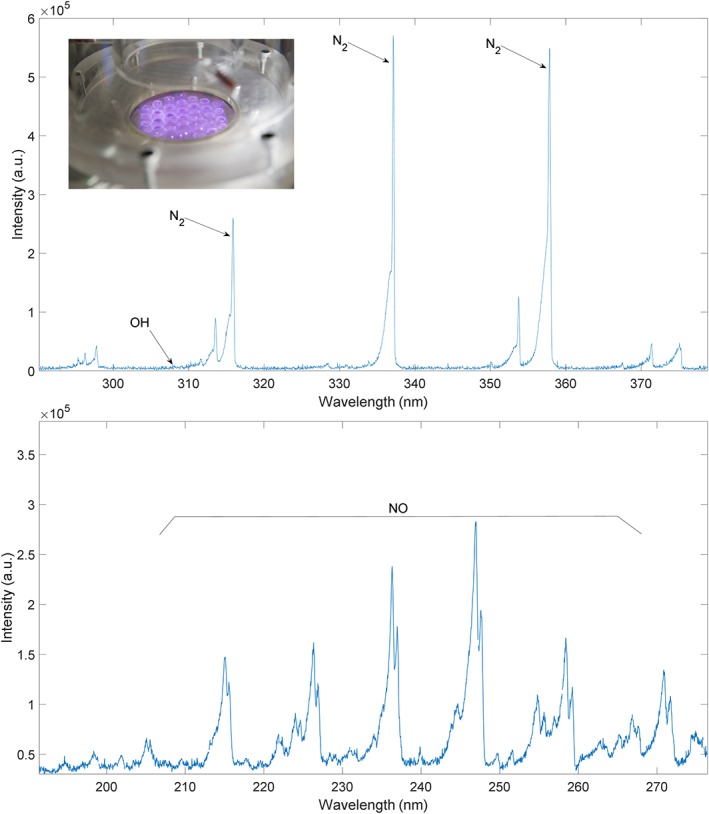
Spectral analysis of the plasma with air as the input gas. [Color figure can be viewed at http://wileyonlinelibrary.com]

The emission spectrum from the plasma, especially the UV range, was analyzed to provide an insight into the species generated by the plasma as well as to investigate the possibility of photochemical reactions in the liquid near the gas‐liquid interface. Figure 4 shows that emission at a range of wavelengths from UVA to UVC is produced from the DBD plasma for an air feed at atmospheric pressure. The UVC emitted from the plasma is of particular interest as it could provide two pathways for pretreatment. First, UV incident on the gas‐liquid interface will facilitate AOP reaction pathways to produce ^•^OH radicals from H_2_O_2_ and O_3_ already dissolved in the liquid.^57^ As ^•^OH radicals have a higher oxidation potential than H_2_O_2_ and O_3_ (see Table 1), and have a greater potential to delignify biomass, UVC is a desirable by‐product from the plasma. Second, UVC transmitted through the gas‐liquid interface and reaching biomass will provide a direct effect,^58^ but this effect could be trivial as the intensity drops sharply away from the interface.

### *Temperature, pH, and conductivity*

**Figure 5:**
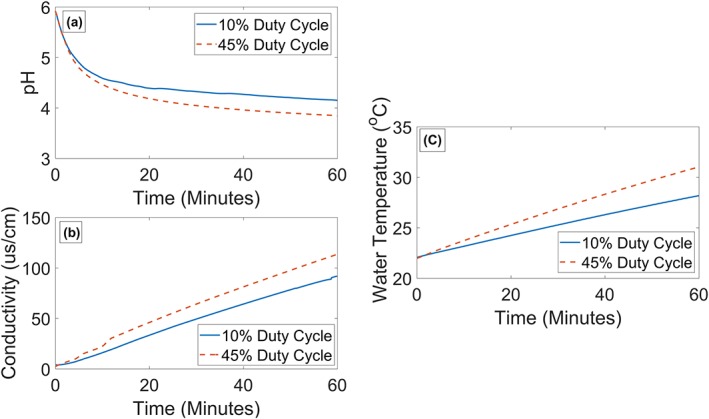
(a) pH (b) conductivity (c) liquid temperature within the pretreatment reactor for 10% and 45% duty cycles. [Color figure can be viewed at http://wileyonlinelibrary.com]

An advantage of forming the plasma adjacent to the liquid phase is convection cooling of the ground electrode by the liquid flow. Figure 5c shows a near linear temperature rise in the liquid phase for both duty cycles during a 60 min operation. As water has a high thermal capacity and is in direct contact with the nickel electrode, liquid in the reactor helps to maintain a low temperature in the plasma module. The 45% duty cycle shows a liquid temperature rise of ∼9°C while 10% duty cycle causes less than 6°C rise after 60 min of operation. As the production and solubility of O_3_ reduces with temperature, significant increase in reactor liquid temperature is not desirable. Therefore, if higher duty cycles were to be used, additional cooling should be considered.

Conductivity and pH influence the solubility of O_3_ and H_2_O_2_ in water. Park et al.,^59^ observed increased levels of dissolved O_3_ and H_2_O_2_ in liquids with higher conductivity, and Dehouli et al.,^60^ saw an increase in O_3_ decomposition as the pH increased from 3 to 7. Figures 5a, b show pH and conductivity respectively in the liquid phase for the two regimes identified previously. Both duty cycles caused an exponential drop in pH with time, where the higher duty cycle acidified the water to a pH of 3.8 as opposed to a pH of 4.2 at 10% duty cycle. Also, both duty cycles led to a linear increase in conductivity with time as ions were continuously introduced to the liquid from the plasma. For both duty cycles, the change in pH and conductivity were of similar magnitude, but the higher duty cycle caused a slightly higher rate of pH drop and increase in conductivity. These trends can be associated with RNS produced by the plasma.^27^ The drop in pH and the increase in conductivity observed here are advantageous as they increase the solubility of O_3_ and H_2_O_2_.

### *Dissolved oxidative species*

**Figure 6:**
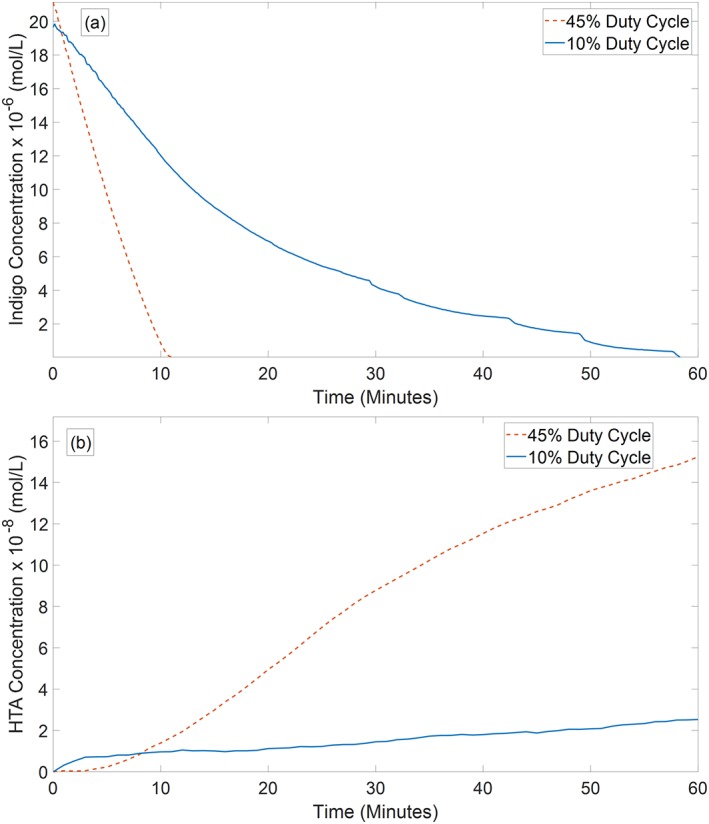
(a) Time evolution of the concentration of Potassium indigotrisulfonate dye for 10% and 45% duty cycle plasma treatments (b) Time evolution of the HTA concentration reflecting the direct and indirect production of ^•^OH for 10% and 45% duty cycle plasma treatments. [Color figure can be viewed at http://wileyonlinelibrary.com]

Figure 6a shows the time evolution of the indigo dye concentration for the two operating regimes identified earlier. The decrease in dye concentration is related to the generation and transfer of oxidative species into the liquid phase of the reactor. It is clear that 45% duty cycle is found to be more efficient in delivering oxidative species as complete breakdown of the dye occurs within 11 min compared to 58 min for 10% duty cycle. This result may seem counterintuitive as production of ozone (an oxidative species well‐known to react with indigo) was found to be most efficient at 10% duty cycle in the empty reactor. A number of factors, however, contribute to this result. The enhanced cooling of the plasma module introduced by liquid circulating over the membrane might have facilitated a lower plasma temperature, which favors O_3_ production. In addition, induced changes in pH and conductivity (Figure 5) increase the solubility of O_3_ into the liquid phase.^59^ And finally, indigo can be oxidized not only by ozone but also by a number of other reactive species produced in the plasma (e.g., ^•^NO, O, ^•^OH, H_2_O_2_), and therefore the degradation rate depends not only on ozone production rate but on the overall reactivity created by the plasma

The HTA production rate under the two operating regimes is shown in Figure 6b, which directly corresponds to the hydroxyl radical concentration in the liquid phase.^49^ OH radical production in the reactor can be attributed to three mechanisms: direct ·OH formation in the plasma, reactions among dissolved plasma species forming ·OH in the liquid phase and conversion of dissolved O_3_ and H_2_O_2_ to ·OH at the gas‐liquid interface in the presence of UVC. It is highly possible that the main contribution comes from the latter mechanisms as ^•^OH in the gas phase will react immediately and its concentration will decrease rapidly away from the active plasma region. If this assumption is valid, ^•^OH radical production should be closely related to plasma species dissolved in the liquid phase. O_3_, superoxide (O_2_^−^) and hydrogen peroxide (H_2_O_2_) are relatively long‐lived species that can be dissolved in the liquid phase and can contribute to ^•^OH radical production. As shown in Figure 6b, 45% duty cycle results in a higher rate of OH production and the final HTA concentration after 60 min is four times higher than that of 10% duty cycle. This result demonstrates the suitability of higher duty cycles for ^•^OH production.

### *Miscanthus compositional analysis*

The results of polymeric sugar and acid soluble lignin (ASL) analysis for miscanthus grass are shown in Table 2. This result is comparable to the compositional analysis provided by Arnoult and Brancourt‐Hulmel^4^ and Li et al.,^5^ which reported 40.6–46.4% of glucan, 28.8–29.2% of hemicellulose, and 19.3–31% of lignin for miscanthus. Slight variations in compositions reported by various authors can be attributed to seasonal variation within plant material and the existence of a variety of miscanthus species around the world.^61^, ^62^

**Table 2:** Sugars and Lignin Analysis of Milled Sieved Miscanthus (% g/g Dry Matter)

ASL	Total Lignin	Glucan	Xylan	Arabinan	Total Sugars
0.153	24	42.07 ± 1.2	15.59 ± 1.1	4.53 ± 0.5	62.18 ± 2.82

### *Acid soluble lignin and sugars released*

**Figure 7:**
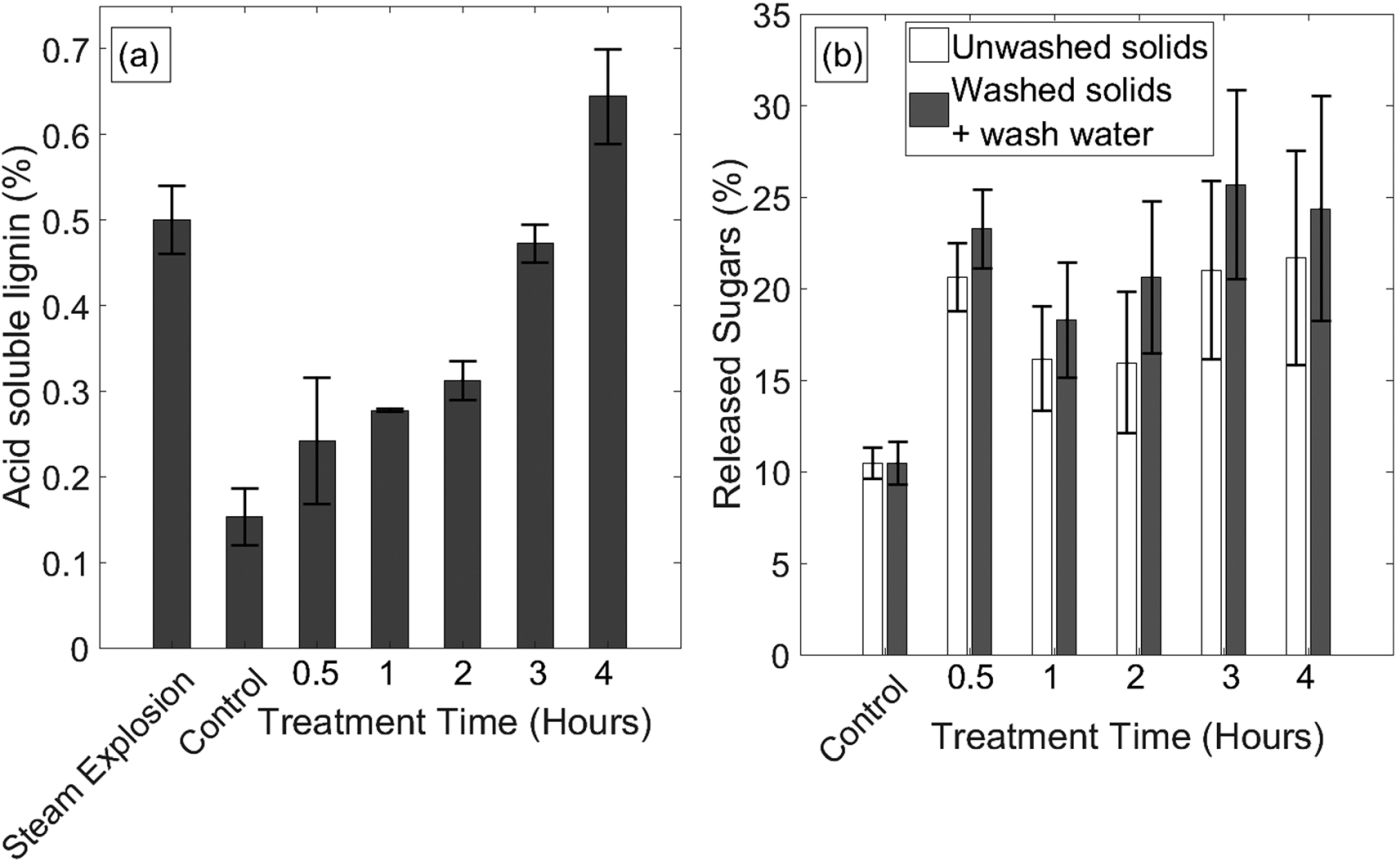
(a) Effects of pretreatment time on ASL release from miscanthus. The duty cycle was fixed at 10% and 5% (wt/wt) miscanthus suspensions were used. (b) Effects of pretreatment time on total sugars released after enzymet hydrolysis. Miscanthus concentration and duty cycle were fixed at 5% and 10%, respectively. “Washed solids + wash water” include the sugars in wash water prior to hydrolysis.

The effectiveness of the pretreatment, that is, to remove or loosen up the lignin structure and to provide access to polymeric sugars for enzymatic hydrolysis, was investigated by measuring ASL release after pretreatment and sugar release after hydrolysis for various operating conditions. With the reactor investigated in this study, ROS and RNS produced by the plasma attacked the biomass and released lignin into the solution causing an increase in measured ASL. The treatment time was varied from 0.5 h to 4 h for 5% (wt/wt) miscanthus solutions, and the ASL released (percent of total biomass dry weight) is shown in Figure 7a. The duty cycle was fixed at 10% for this case to minimize membrane damage from long exposure to the DBD plasma. The amount of ASL released increased with increasing pretreatment time giving a clear indication that the plasma microbubble reactor is able to cause lignin degradation. The ASL release shows a relatively slow increase over the first 2 h of treatment (∼0.075% ASL release per hour), with more rapid release from 2 to 4 h (∼0.175% ASL release per hour) showing a pattern of late acceleration. However, enzymatic sugar release shows a different trend: a significant increase in the first 0.5 h followed by a plateau. The sugars released by enzymatic hydrolysis after various pretreatment times varied between 18% and 26% as shown in Figure 7b. This suggests that reactive species may initially be targeting something which releases a significant proportion of sugars, at the start (e.g., ferulic acid, or the waxes that may be present), and only after these have been released does a more rapid degradation of the lignin occur. Furthermore, the lignin breakdown had not plateaued after 4 h indicating the possibility of further degradation with time. Pretreatment may be accelerated by operating the reactor with intensified conditions, but additional cooling of the plasma will be required to maintain a constant rate of ozone production for longer periods. Moreover, it may be possible to shorten the treatment time by further size reduction of miscanthus to improve contact between miscanthus and reactive species.

These figures are comparable to the values reported for sawdust after ozonolysis.^63^ In addition to ozone treatment, this study used a fungal culture (*Phanaerochaete chrysosporium*) following by ozonolysis to improve digestibility of miscanthus from 12.4% to 26.9%. The level of sugar release for the control experiment without any pretreatment is also comparable to the 9% sugar yields obtained by Huang et al.,^64^ Panneerselvam et al. reported pretreatment of miscanthus in a packed bed reactor using pure ozone generated from an ozone generator.^65^ The ozone concentration was varied from 40 to 58 mg/L, and up to 60% lignin was removed with minimal cellulose degradation. However, sugar conversion reported with pretreated material was relatively low compared to raw miscanthus due to enzyme inhibition caused by degradation products released during pretreatment. Sannigraghi et al., have also reported relatively low sugar yields of 4–10% for ozone treatment.^66^ However, when a two‐step pretreatment was applied to miscanthus, that is, ozone treatment followed by autohydrolysis at high temperatures, sugar yields were increased to 68%. These studies indicate that further improvements to sugar release are possible by combining ozone pretreatment with a secondary treatment method such as heat treatment. The steam pretreated miscanthus samples in our study yielded 65%–78% sugars under the same enzymatic hydrolysis conditions. This suggests that even though ozonolysis can help to increase sugar yields from lignocellulosic materials, a combined approach of ozonolysis and steam pretreatment might be more effective as described by Sannigraghi et al.^66^ However, this will be advantageous only if the combined approach either releases more sugars or is less energy intensive than steam explosion on its own. Even though low sugar yields with ozone pretreatment have been widely reported,^63^, ^64^, ^65^, ^66^ several studies have shown better enzymatic hydrolysis following ozonolysis. Vidal and Molinier,^67^ reported that ozonolysis of poplar sawdust decreased the lignin content from 29% to 8% leading to an increase in sugar yield from 0% to 57%. Garcia‐Cubero et al.^21^ observed improvement of sugar yields from 29% to 88.6% for wheat straw and 16% to 57% for rye straw following ozonolysis.

To determine the effect of washing on pretreated samples prior to enzymatic hydrolysis, separate washed samples were also analyzed including the wash water. Figure 7b indicates that washing improves sugar release by up to ∼5% when considered with the sugars removed by the wash water. These results suggest that the ASL released during ozone pretreatment may inhibit enzymatic hydrolysis and washing the samples before hydrolysis could improve the effectiveness of hydrolysis. Sannigraghi et al. have shown that ozone pretreatment followed by washing of miscanthus will reduce the sugar content of the ozone treated biomass, if the sugars removed by washing are not recovered.^66^ They found 0.1% to 9% (wt/wt) of sugar removal with wash water. Bono et al., have also shown that washing ozone treated samples leads to a significant increase in sugar hydrolysis as shown in this study.^63^

The relatively low hydrolysis yields encountered in this study may be attributed to enzyme inhibition caused by degradation products of miscanthus, reactive species attacking sugars during and after pretreatment or the lack of adequate mixing during ozonolysis. Although ozone pretreatment is largely reported as not producing inhibitory/toxic products that could affect enzymes or fermentation bacteria, the nature of degradation products depends on the biomass type and the plasma products dissolved in the pretreatment liquid. Bellido et al.^68^ suggested that the presence of inhibitory compounds such as oxalic acid and acetic acid from ozone pretreatment could inhibit fermentation of unwashed wheat straw hydrolysates by *P. stipitis*. Cellulolytic enzymes may also be inhibited by the plasma products dissolved during pretreatment but washing the pretreated biomass can reduce this inhibition.^65^ To determine such inhibitory effects, fermentability of the pretreated samples was tested using C5 fermenting yeast (DSM) and *Geobacillus thermoglucosidasius* (ReBio LTD, UK) by fermenting the post enzyme hydrolysis‐treated miscanthus for 48 h. Ethanol yields of 95%–98% were obtained indicating no toxicity to the fermenting organisms. Pretreated biomass solutions were stored frozen with plasma activated liquids for approximately 2–3 weeks prior to hydrolysis and fermentation; therefore, it is possible that ROS and RNS could have degraded some sugars following pretreatment. ROS such as ^•^OH radicals are nonselective and could react with sugars.^69^ This effect could be mitigated by washing the treated samples immediately after the ozone microbubble pretreatment.

**Figure 8:**
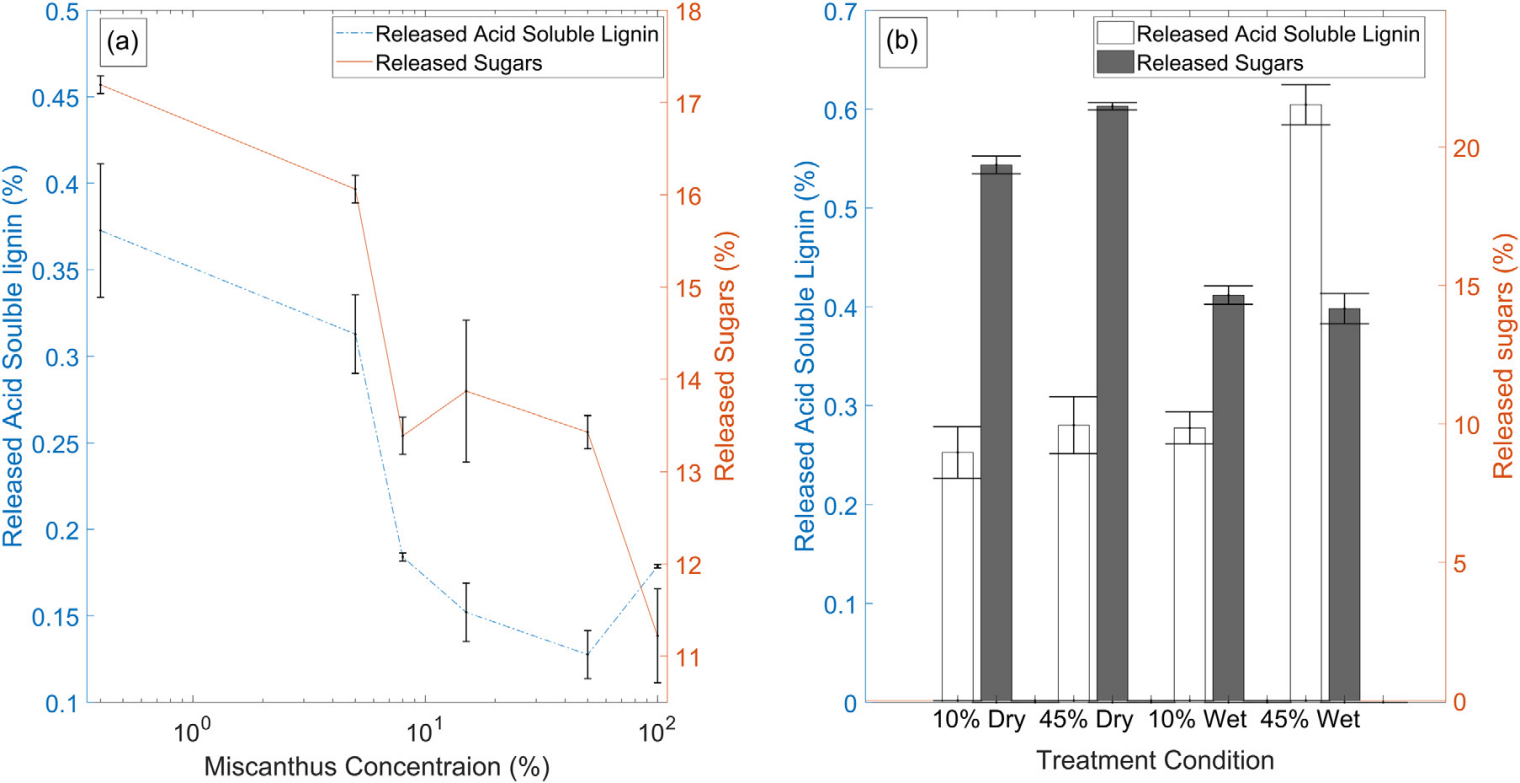
(a) Effects of miscanthus concentration on ASL and sugars released. Duty cycle was fixed at 10% and the treatment time is 2 h. (b) ASL release for miscanthus particles at equilibrium moisture content (dry) and a 5% (w/w) solution in water (wet) at 10% and 45% duty cycles. The miscanthus concentration and treatment time was fixed at 5% (wt/wt) and 1 h. [Color figure can be viewed at http://wileyonlinelibrary.com]

Figure 8a shows the ASL and sugars released with varying concentration of miscanthus in water for 10% duty cycle and 2 h plasma microbubble treatment. It can be seen that the concentration of biomass has a significant effect on ASL released and sugars released, with dilute solutions leading to higher lignin removal and high sugar release. Solutions of low biomass concentrations are relatively easy to mix by microbubble clouds as opposed to highly viscous solutions made of high biomass concentrations. In addition to this, the consumption of dissolved reactive species will also depend on miscanthus concentration in water. However, the amount of lignocellulosic biomass treated in a fixed volume reactor decreases with concentration; hence a trade‐off must be found. Mixing and contact between reactive species and miscanthus can be improved using mechanical stirring or further size reduction of biomass, but the additional energy requirement should be sufficiently low to retain the benefits achieved with pretreatment efficiencies.

A comparison of ASL released for dry vs. wet ozonolysis pretreatment is shown in Figure 8b. The duty cycle used was either 10% or 45% for miscanthus with equilibrium moisture content and a 5% (wt/wt) solution in water. The pretreatment time was kept constant at 1 h for these experiments. Even though, only a marginal difference in ASL release is observed between wet and dry treatment for 10% duty cycle, a significant effect can be seen at 45% duty cycle. The ASL released for wet treatment at 45% duty cycle doubled compared to the dry treatment at the same duty cycle indicating the effectiveness of wet treatment. However, sugars released post hydrolysis for the dry treatments were higher than that for the wet treatments. This is an interesting observation as one would expect higher sugar release to be associated with high ASL release. This could be an indication that the residual ROS and RNS in the liquid postpretreatment are detrimental to the released sugars. SEM micrographs were analyzed to understand the effects of moisture content on ozonolysis pretreatment.

### *Scanning electron microscopy*

**Figure 9:**
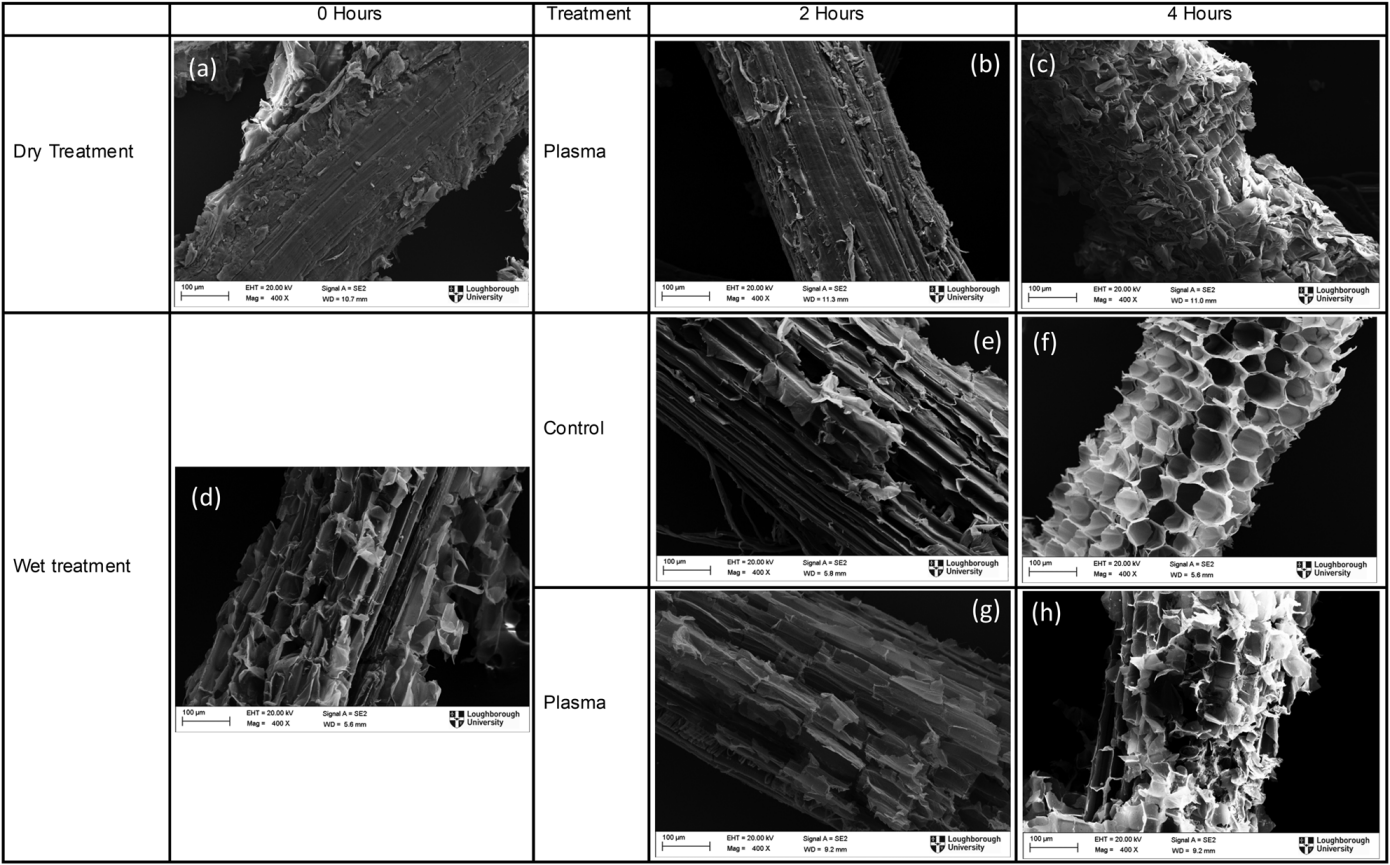
SEM micrographs of miscanthus samples for wet and dry pretreatment. (a) dry miscanthus without any pretreatment (b) fluidized bed treatment after 2 h (c) fluidized bed treatment after 4 h (d) miscanthus in water immediately after soaking (e) miscanthus in water after 2 h without pretreatment (f) miscanthus in water after 4 h without pretreatment (g) plasma microbubble pretreated miscanthus after 2 h (h) plasma microbubble pretreated miscanthus after 4 h.

SEM micrographs allow observation of structural changes to miscanthus before and after the pretreatment at various operating conditions. Images were taken for both the wet and dry treatments after 0, 2, and 4 h treatments with a 45% duty cycle. The dry miscanthus structure shown in Figure 9a is highly dense indicating limited surface exposure to the gases produced by the plasma. Slight structural damages are visible at 2 h of dry pretreatment as seen in Figure 9b, with significant localized damage appearing after 4 h of treatment. The structure after soaking miscanthus for 1 h is shown in Figure 9d. Clearly, miscanthus in water swells to give a large surface area and enables greater penetration of the reactive species (Figures 9e, f). The images shown in Figures 9g, h indicate destruction of the structure due to plasma microbubble pretreatment compared to the relevant control tests (Figures 9e, f). Clearly, longer treatment times lead to more structural damage to miscanthus, and there is a strong agreement between the ASL released shown in Figure 7a and SEM micrographs.

### *Power consumption*

A key design criterion for the pretreatment reactor is to maximize production of selective oxidative species and to transfer them effectively to the liquid phase with the least energy input. The power consumption of the plasma power source was found to increase linearly with the duty cycle and consumed 6.9 W and 18.6 W for 10% and 45% duty cycles, respectively. However, plasma power was only a fraction of the power drawn, hence the power source efficiencies were calculated as 30% for 10% duty cycle and 48% for 45% duty cycle. To estimate the power consumption based on the treatment effect and to compare with steam explosion, energy required to release 1 kg of ASL was calculated from the experimental data acquired. The plasma microbubble reactor consumed 0.4 kWh/kg and 0.3 kWh/kg at 10% and 45% duty cycles, respectively, while steam explosion consumed 8.2 kWh/kg. This is a significant improvement in terms of energy usage for pretreatment, but further study is warranted to improve sugar yields to make this approach feasible. The energy used in producing compressed air for microbubbles and mechanical mixing in steam explosion reactor were not included in this calculation as these costs will not increase linearly with scaled‐up operation. The power supply was designed for versatility rather than energy efficiency and there is significant room for improvement. Nonetheless, the efficiency of the power supply unit was considered in calculations above when comparing treatment efficiency with steam explosion.

(1)

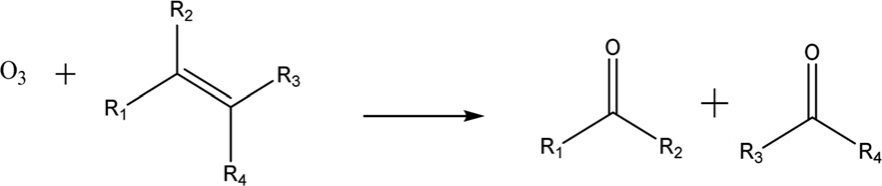

(2) $$\text{duty cycle}\;(\%)=\left(\frac{‘on^{}’time}{‘on’time+‘off’time}\right)*100$$

(3)

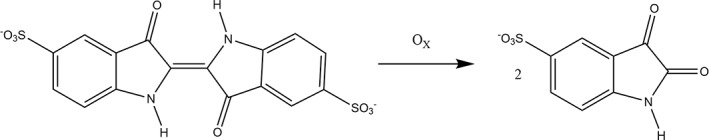

(4)

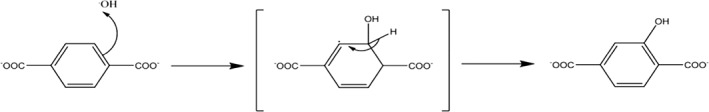

(5) $$ASL\left(\%\right)=A\times D\times V\times K\times W\times 100$$

## Conclusions

In converting lignocellulosic biomass to biofuels, pretreatment plays a key role in process economics; hence energy efficient and cost‐effective pretreatment methods are required to make the process sustainable and financially viable. Ozonolysis has been used for pretreatment with limited success, but to make the full use of short‐lived reactive species produced by atmospheric air plasma, a novel design is required to bring the plasma to the gas‐liquid interface. Additionally, the reactor should not be mass transfer limited to take the full advantage of the gaseous products formed by the plasma. For this purpose, a novel plasma microbubble reactor has been designed and tested for pretreatment of miscanthus grass.

A range of ROS and RNS were generated by the plasma and transferred to the liquid phase via microbubbles to remove lignin in miscanthus solutions. The duty cycle of the power source was identified as a key operating parameter that controlled the plasma temperature and thereby the composition of the cocktail of reactive species generated by the plasma. Two operating regimes were identified for the empty reactor. The low duty cycles (∼10%) were found to favor O_3_ production while the high duty cycles (∼45%) promoted production of reactive nitrogen species. A maximum ozone concentration of 426 ppm was recorded for the 10% duty cycle after 15 min of operation. However, addition of liquid to the reactor and consequent cooling of the plasma module allowed high duty cycles to be used with a greater generation rate of O_3_ and ^•^OH radicals compared to operation at low duty cycles. Spectral analysis confirmed emission of UVC from the plasma that would participate in producing hydroxyl radicals at the gas‐liquid interface via photochemical reactions.

The effectiveness of the plasma microbubble treatment was assessed by measuring acid soluble lignin released after pretreatment and sugars released after enzymatic hydrolysis. Low miscanthus concentrations of ∼5% (w/w) were pretreated more effectively than highly concentrated solutions as mixing intensity decreases with biomass loading. Increasing the pretreatment time increased the acid soluble lignin released; with slow release from 0 to 2 h followed by an accelerated release from 2 to 4 h. The acid soluble lignin released after a 4 h treatment was 0.65% (w/w) demonstrating the effectiveness of this approach. Sugars released posthydrolysis varied between 18% and 26% with a rapid release within the first 0.5 h followed by a plateau. Accelerated pretreatment observed toward the end of a 4 h pretreatment indicates the requirement for longer pretreatment times or more intensified treatments. Based on the outcomes of this study, a further investigation is recommended with the following modifications to achieve the full potential of this plasma‐microbubble approach. (1) Improved cooling of the plasma to avoid a drop‐in ozone generation (2) Further size reduction of miscanthus to improve mass transfer and mixing (3) Neutralization and washing of miscanthus immediately after pretreatment to avoid sugar degradation due to reactive species in plasma activated water (4) longer pretreatment runs to identify the optimum pretreatment time and maximum potential of this approach.
